# Artificial intelligence-assisted diagnosis of early allograft dysfunction based on ultrasound image and data

**DOI:** 10.1186/s42492-025-00192-z

**Published:** 2025-05-12

**Authors:** Yaqing Meng, Mingyang Wang, Ningning Niu, Haoyan Zhang, Jinghan Yang, Guoying Zhang, Jing Liu, Ying Tang, Kun Wang

**Affiliations:** 1 CAS Key Laboratory of Molecular Imaging, Institute of Automation, Chinese Academy of Sciences, Beijing, 100190 China; 2 School of Artificial Intelligence, University of Chinese Academy of Sciences, Beijing, 101408 China; 3 Department of Ultrasound, Tianjin First Central Hospital, NanKai University, Tianjin, 300192 China

**Keywords:** Liver transplantation, Early allograft dysfunction, Artificial intelligence, Ultrasound

## Abstract

Early allograft dysfunction (EAD) significantly affects liver transplantation prognosis. This study evaluated the effectiveness of artificial intelligence (AI)-assisted methods in accurately diagnosing EAD and identifying its causes. The primary metric for assessing the accuracy was the area under the receiver operating characteristic curve (AUC). Accuracy, sensitivity, and specificity were calculated and analyzed to compare the performance of the AI models with each other and with radiologists. EAD classification followed the criteria established by Olthoff et al. A total of 582 liver transplant patients who underwent transplantation between December 2012 and June 2021 were selected. Among these, 117 patients (mean age 33.5 ± 26.5 years, 80 men) were evaluated. The ultrasound parameters, images, and clinical information of patients were extracted from the database to train the AI model. The AUC for the ultrasound-spectrogram fusion network constructed from four ultrasound images and medical data was 0.968 (95%CI: 0.940, 0.991), outperforming radiologists by 30% for all metrics. AI assistance significantly improved diagnostic accuracy, sensitivity, and specificity (*P* < 0.050) for both experienced and less-experienced physicians. EAD lacks efficient diagnosis and causation analysis methods. The integration of AI and ultrasound enhances diagnostic accuracy and causation analysis. By modeling only images and data related to blood flow, the AI model effectively analyzed patients with EAD caused by abnormal blood supply. Our model can assist radiologists in reducing judgment discrepancies, potentially benefitting patients with EAD in underdeveloped regions. Furthermore, it enables targeted treatment for those with abnormal blood supply.

## Introduction

Liver transplantation (LT) significantly improves the survival rate and quality of life of patients with end-stage liver disease [1]. Advances in medical techniques have increased LT success rates to 80%–90% [2]. The current focus has shifted towards enhancing patient prognosis and long-term survival rates [3]. However, early allograft dysfunction (EAD) remains a common post-LT complication, affecting approximately 20%–40% of recipients [4, 5]. EAD is often correlated with poor prognosis and high mortality. Studies indicate that 63% of patients experiencing early post-LT graft loss develop EAD, resulting in significantly lower survival rates than those without EAD [6, 7]. Additionally, patients with EAD are prone to other organ injuries, further affecting their long-term prognosis [8, 9].

The etiology of post-LT EAD is complex, and current diagnostic criteria rely solely on laboratory indicators, which are insufficient to determine its specific cause [10–13]. Additionally, effective noninvasive imaging techniques for diagnosing EAD remain limited [14–17]. Blood supply to the graft during the early post-LT stages is crucial for graft function recovery [18, 19]. Therefore, it is essential to explore whether abnormal hemodynamics in the transplanted liver are independent factors influencing the occurrence of EAD and whether such abnormalities can be detected through imaging.

LT surgery involves the reconstruction of multiple blood vessels, making smooth blood flow in the transplanted liver essential for graft survival. Stable hemodynamics are key to ensuring the recovery of graft function. Although the pathophysiological mechanisms underlying EAD remain unclear, some studies have suggested that its occurrence may be related to ischemia/reperfusion injury [20, 21]. This injury is characterized by a microcirculatory blood flow disorder that further affects the hemodynamic status of the transplanted liver. Thus, analyzing the hemodynamics of the transplanted liver is crucial for EAD diagnosis.

Ultrasound is a noninvasive, real-time bedside imaging method used for early blood flow detection post-LT. It can measure the intravascular flow velocity, systolic and diastolic phases, and blood flow spectrum using pulsed-wave Doppler, making it particularly suitable for detecting blood flow abnormalities [22, 23]. As a real-time dynamic imaging technique, ultrasound can quantitatively evaluate the hemodynamic status of the transplanted liver through color Doppler technology, providing various quantitative hemodynamic parameters, such as blood flow velocity and resistance index. It enables repeated, noninvasive perioperative monitoring of graft blood flow changes in real time post-LT, making it the preferred method for assessing graft hemodynamics [24–26].

Deep neural networks can quantify imperceptible image features in a high-throughput manner [27–29], enhancing liver diseases diagnosis accuracy [30, 31]. However, deep learning analysis of ultrasound images has not yet been applied to LT [32]. Previous studies on liver diseases using deep learning have focused on grayscale, elastic graphics, and Doppler ultrasound without analyzing flow spectrograms from Doppler ultrasound. The ResNeXt architecture was selected for its efficient feature extraction capabilities and superior performance, demonstrating robustness in other ultrasound imaging classification tasks [33, 34].

Based on these insights, the objectives of this study are as follows:

- To develop a deep learning model for the quantitative analysis of ultrasound blood flow spectrograms of the liver parenchyma, hepatic veins, portal veins, and hepatic arteries and validate its diagnostic accuracy for EAD.
- To assess whether deep learning can effectively assist radiologists in improving the diagnosis of EAD.
- To investigate the correlation between EAD occurrence and blood flow abnormalities by comparing the diagnostic efficacy of deep learning models using ultrasound images of the liver parenchyma and the three major blood vessels.

## Methods

### Study design

This retrospective study was approved by the Medical Ethics Committee of Tianjin First Central Hospital (ethics approval number: 2021N058KY). We reviewed patients who underwent LT at our hospital between December 2012 and June 2021. The patients were randomly categorized into training and validation cohorts in a ratio of 5:1. The training cohort was used to train network models such as the ultrasonic spectrum fusion network, whereas the validation cohort was used to validate the model performance and compare the diagnostic accuracy between the models and radiologists.

### Patient enrollment

The inclusion criteria were:

1. Patients undergoing LT (3 months to 77 years old); (2) patients undergoing ultrasound examination 1–7 days after LT; (3) laboratory data 1–7 days post-surgery, allowing EAD diagnosis based on standard criteria; (4) patients with complete ultrasound and general information data; and (5) those whose ultrasound images meet the annotation standards.

By contrast, the exclusion criteria were:

1. Patients who did not undergo ultrasound examination 1–7 days after LT; (2) patients whose ultrasound data or general conditions of the patient are missing; (3) those whose ultrasound images do not meet the labeling standards.

### Bedside ultrasound examinations

Ultrasound images, liver parenchyma and blood flow parameters were obtained according to the Chinese Technical Specifications for Ultrasound Imaging Diagnosis and Treatment of Organ Transplantation (2019 edition) [35]. The collected patient images span a long period, including various machine models and operators, which helps mitigate data source variations in model training.

Images captured for each patient included: (1) grayscale ultrasound images of the liver parenchyma, (2) color Doppler ultrasound images of portal vein, the velocity of portal vein, (3) color Doppler ultrasound images of the hepatic arteries, the peak systolic velocity, and the end-diastolic velocity of the hepatic arteries, (4) color Doppler ultrasound image of hepatic vein, the velocity of hepatic vein.

All flow velocity measurements were taken three times and averaged.

The ultrasound diagnostic instruments used were Acuson S2000, Acuson Sequoia512, GE Logic E9, Philips iU22, Mindray DC80, and Mindray M7 bedside ultrasound diagnostic instruments with convex array probes (frequency 3–5 MHz).

After image acquisition, radiologists reviewed them based on strict inclusion and annotation criteria. Initially, a radiologist determined whether each patient’s image met the established criteria. Specifically, grayscale ultrasound images had to be clear and stable without measurement lines or artifacts and must clearly depict the liver parenchyma structure. Doppler ultrasound images had to be clear and stable, with a blood flow spectrum clearly displaying at least three consecutive cardiac cycles.

Conversely, certain cases may involve ultrasound images that are challenging to annotate owing to irregular operational procedures, unclear scanning or acquisition, or the presence of measurement lines or markers that interfere with the delineation of the region of interest. Such images did not meet the annotation criteria and were excluded.

Subsequently, the radiologists annotated the grayscale ultrasound images of the liver parenchyma and blood flow spectrograms in the Doppler ultrasound images.

For the liver parenchyma, a grayscale ultrasound image with a clear and stable depiction of the liver structure was selected. The region of interest was placed within the liver parenchyma to select an area that was structurally complete and devoid of measurement lines or artifacts. The region of interest was then adjusted to include the largest portion of the liver parenchyma before annotation and labeling.

The image with a clear and stable spectrum was selected as the liver blood flow spectrum. The region of interest was centered on the baseline, ensuring symmetry on both sides of the baseline. The hepatic artery spectrum required a complete cardiac cycle, whereas the portal and hepatic vein spectra required two consecutive complete cardiac cycles.

### AI models

#### Data preprocessing

The model inputs were divided into imaging and clinical data. The image inputs included grayscale ultrasound images, portal vein blood flow spectrograms, hepatic artery blood flow spectrograms, and hepatic vein blood flow spectrograms. Clinical data included portal vein velocity, peak systolic velocity, end-diastolic velocity, and hepatic vein velocity.

Images were converted to grayscale, resized to uniform dimensions, and standardized using the Z-scoring method. Data augmentation included random rotation and random perspective transformation to enhance the generalization of the model and reduce overfitting. Clinical data were normalized using the min-max method and transformed into dimensionless values.

#### Deep learning neural network

**Fig. 1:**
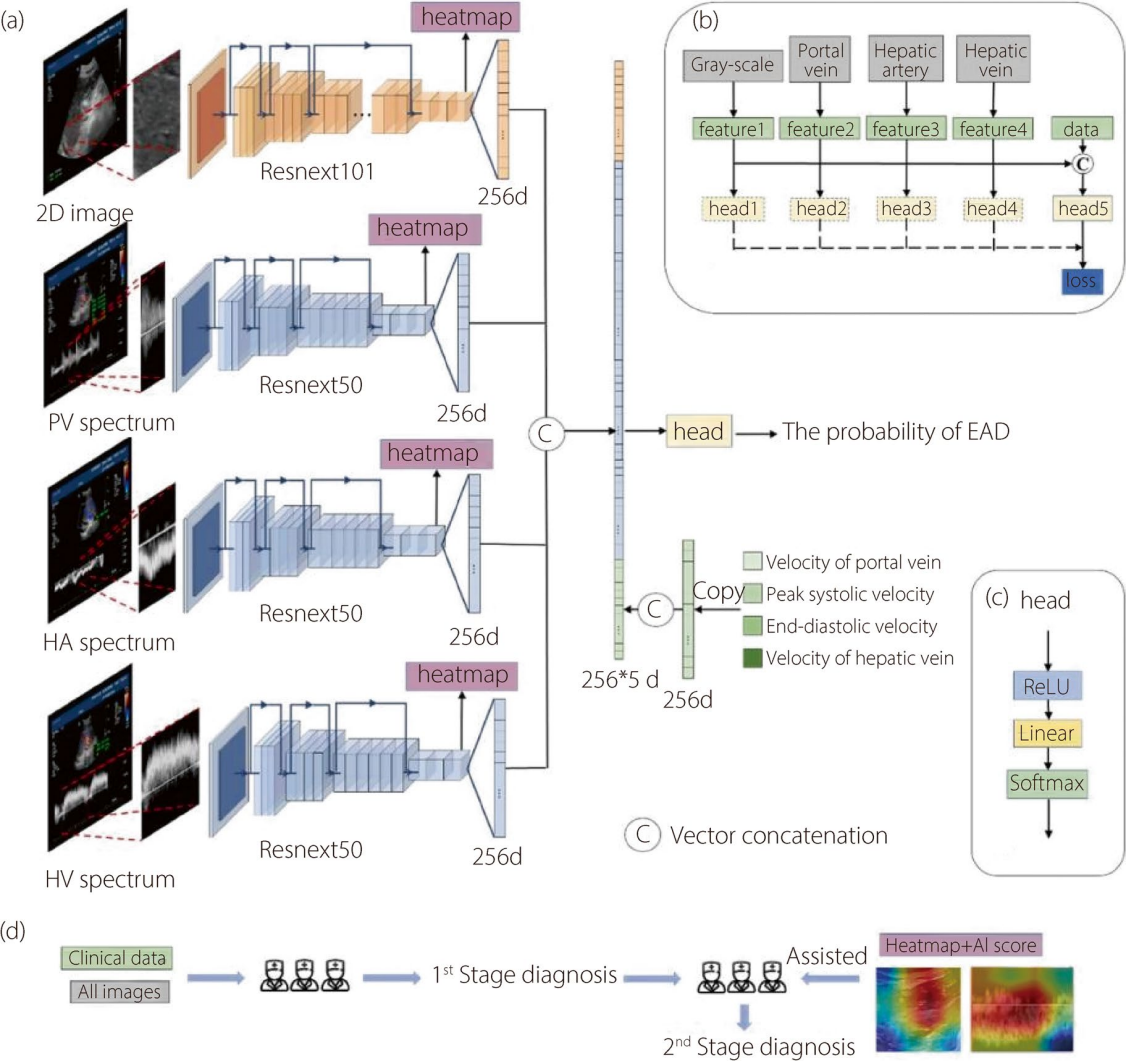
Illustration of the ultrasound-spectrogram fusion network. **a** Architecture of a network with four images and clinical data as inputs and EAD probability as the output (2D: Grayscale ultrasound image, PV spectrum: Portal vein blood flow spectrum diagram, HA spectrum: Hepatic artery blood flow spectrum diagram, HV spectrum: Hepatic vein blood flow spectrum diagram); **b** Network training and validation strategies; **c** Classification head structure; **d** Flowchart of the two-stage study

Four types of images and clinical data were input into the ultrasonic spectrum fusion network for multichannel image fusion and analysis. The backbone network extracted feature maps from all four images. For grayscale ultrasound images, which contained more information, a deeper network (ResNeXt101) was employed, while shallower networks (ResNeXt50) were applied to the three spectral images [36]. Using global average pooling and fully connected layers, 256-dimensional feature vectors were obtained for each image. The clinical data were replicated 64 times to form a 256-dimensional feature vector. These feature vectors were concatenated, and a fully connected classification head was used to classify the data, outputting the probability of EAD (Fig. 1).

#### Training and validation

In the training phase, an auxiliary fully connected classification head was assigned to the feature vector of each image input. The cross-entropy loss was calculated between the gold label and the output of these heads, including the final head, to optimize the model [30]. In the validation phase, auxiliary classification heads and data augmentation methods were excluded. Layer-CAM was applied to the final stage feature maps of the feature extractors to visualize the heat maps [33, 34].

### EAD diagnosis

The diagnosis of the EAD group was based on the criteria established by Olthoff et al.: (1) Total bilirubin ≥ 10 mg/dL on the seventh postoperative day. (2) International normalized ratio ≥ 1.6 on the seventh postoperative day. (3) Aspartate aminotransferase or alanine aminotransferase > 2000 U/L within seven days. Meeting any of the above criteria qualified a patient for an EAD diagnosis.

### Clinical utility

A two-stage study was conducted to evaluate the diagnostic performance and clinical applicability of the model. Six radiologists participated in the study and were categorized into two groups based on their work experience: seniors and juniors. The senior group included chief physicians with 25 years of experience, associate chief physicians with 11 years of experience, and attending physicians with 10 years of experience in transplant ultrasound. The junior group included an attending physician with six years of experience, another attending physician with five years of experience, and a resident with six years of experience in transplant ultrasound.

#### First phase

The validation cohort was shuffled and administered to both the groups of doctors. Each doctor independently judged whether EAD had occurred based on the grayscale ultrasound image, portal vein blood flow spectrum, hepatic artery blood flow spectrum, hepatic vein blood flow spectrum, and clinical data. The accuracy and other indicators were recorded.

#### Second phase

The model provides doctors with a diagnosis heatmap and EAD probability. Each doctor revised or maintained their initial judgment after reviewing the model output, and the accuracy and other indicators were recorded again.

### Statistical analysis

All statistical analyses were conducted using the SPSS and Python software. Continuous variables are expressed as mean ± SD. Independent sample *t*-tests were used to compare the measurement data between the two groups and *χ*^2^ tests were used for count data comparisons. Receiver operating characteristic (ROC) curve analysis was used to assess the diagnostic performance of the model in both cohorts. Micro-averaging was used to plot multiclass ROC curves. The 95%CI were calculated using a bootstrap method with 100 re-samples. Differences in performance between radiologists of different seniorities and between radiologists with and without AI assistance were assessed using independent-sample *t*-tests. Statistical significance was set at *P* < 0.050 was considered statistically significant.

## Results

### Dataset construction

This retrospective single-center study used the Clinical LT Ultrasound Image Database Software V1.0 (registration number: 2021SR0368364), which was developed independently by our center. A total of 1090 patients who underwent LT at our hospital between December 2012 and June 2021 were selected based on the diagnostic criteria for EAD and normal postoperative outcomes. After applying the inclusion criteria and sorting, 582 patients were included in this study. The ultrasound examination parameters, images, and clinical data of patients were extracted from the database.

### Model performance

**Fig. 2:**
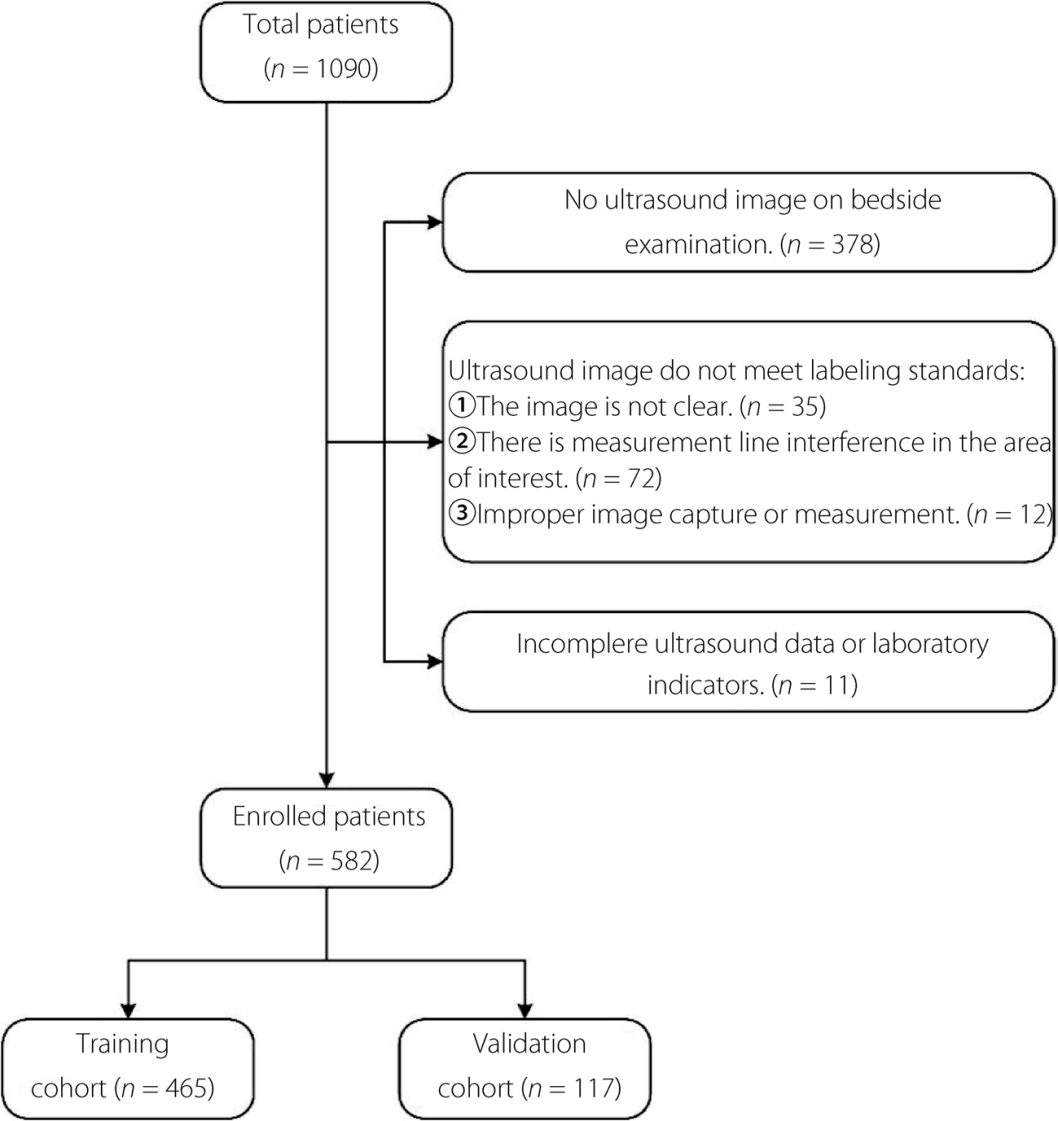
Patient selection flowchart

We included 582 patient records, with 280 patients in the EAD group and 302 in the normal group. To maintain class balance, 485 cases were randomly selected for the training cohort used in model development, whereas the remaining 97 cases constituted the validation cohort to simulate prospective experimental conditions. This validation cohort was also used for doctor-machine comparison. No significant differences in patient characteristics were observed between the training and validation cohorts (Table 1, Fig. 2).

**Table 1:** Characteristics of patients in the training and validation cohorts

Characteristic	Training cohort (*n* = 465)	Validation cohort (*n* = 117)	*P *value
EAD incidence	NA	NA	1.000
EAD	224	56	NA
Non-EAD	241	61	NA
Age (year)	45 (0.83, 57)	45 (0.79, 57)	0.933
Sex	NA	NA	0.826
Male	312	80	NA
Female	153	37	NA
BMI	21.9 ± 4.9	21.9 ± 4.5	0.914
Labortatory MELD score	13 (9, 17)	13 (9, 16)	0.533
Liver disease	NA	NA	NA
Alcohol related disease	36	4	0.106
Viral hepatitis	186	51	0.528
Biliary liver disease	172	48	0.456
Other	46	8	0.375
Acute liver failure	9	3	0.715
Carcinoma	130	35	0.731
Donor type	NA	NA	NA
DBCD	294	73	0.915
LDLT	171	44	NA

*BMI* Body mass index, *MELD* Model for end-stage liver disease, *DBCD* Donation after brain and cardiac death, *LDLT* Living donor liver transplantation, *NA* Not applicable

The training and validation cohorts were used to train and evaluate the ultrasonic fusion network. To explore EAD caused by abnormal blood supply, separate classification heads were trained and evaluated for grayscale ultrasound images, portal vein blood flow spectrograms, hepatic artery blood flow spectrograms, and hepatic vein blood flow spectrograms. Additionally, a model incorporating only three spectrograms and clinical data was designed, trained, and evaluated.

The areas under the curve (AUC) for the models constructed using the four images were as follows:

1. Grayscale ultrasound imaging model: 0.833 (95%CI: 0.769, 0.911)
2. Portal vein blood flow spectrum model: 0.764 (95%CI: 0.692, 0.833)
3. Hepatic artery blood flow spectrum model: 0.838 (95%CI: 0.758, 0.903)
4. Hepatic vein blood flow spectrum model: 0.964 (95%CI: 0.951, 0.977)

The AUC for the model with three spectrograms and clinical data was 0.941 (95%CI: 0.893, 0.980). The AUC for the ultrasound-spectrogram fusion network, which incorporated four images and clinical data, was 0.968 (95%CI: 0.940, 0.991). Ablation experiments were conducted on different data types, and the results indicated that data fusion significantly improved the performance of the model (Table 2).

**Table 2:** AI model metrics

Model	Cohort	AUC	Accuracy (%)	Sensitivity (%)	Specificity (%)
2D model	Training	0.862 (0.832, 0.887)	78.9 (75.7, 81.6)	75.6 (71.5, 79.4)	83.9 (79.7, 87.5)
	Validation	0.833 (0.752, 0.890)	80.0 (72.2, 85.6)	74.5 (65.1, 81.2)	90.3 (79.4, 97.3)
PV model	Training	0.875 (0.847, 0.902)	79.3 (75.7, 82.5)	75.6 (71.5, 79.7)	85.0 (80.4, 88.8)
	Validation	0.756 (0.663, 0.829)	67.3 (58.8, 74.2)	67.2 (55.2, 78.4)	67.7 (55.6, 78.0)
HA model	Training	0.833 (0.803, 0.862)	76.6 (73.6, 79.2)	70.4 (65.9, 74.3)	90.7 (86.7, 94.3)
	Validation	0.763 (0.675, 0.835)	69.9 (62.9, 78.4)	63.9 (55.6, 73.7)	89.0 (77.8, 100.0)
HV model	Training	0.956 (0.935, 0.973)	93.7 (91.1, 95.7)	92.4 (88.8, 95.2)	95.2 (92.3, 97.7)
	Validation	0.879 (0.809, 0.940)	82.4 (75.3, 89.7)	75.7 (66.7, 86.2)	95.2 (88.9, 100.0)
*P* of AUCs for single-input models in the training cohort		< 0.001	*P* of AUCs for single-input models in the validation cohort		< 0.001
Blood supply model	Training	0.994 (0.988, 0.998)	94.3 (92.8, 95.9)	90.2 (87.3, 93.2)	100.0 (100.0, 100.0)
	Validation	0.941 (0.898, 0.973)	84.1 (79.4, 89.7)	76.3 (69.2, 84.3)	100.0 (100.0, 100.0)
USF model	Training	1.000 (1.000, 1.000)	99.6 (99.0, 100.0)	99.6 (98.8, 100.0)	99.5 (98.7, 100.0)
	Validation	0.968 (0.939, 0.987)	85.6 (78.4, 90.7)	92.5 (85.2, 98.1)	79.8 (70.2, 87.5)

2D model: using grayscale ultrasound image

PV model: using portal vein blood flow spectrum diagram

HA model: using hepatic artery blood flow spectrum diagram

HV model: using hepatic vein blood flow spectrum diagram

Blood supply model: using 3 spectrum and clinical data

USF model: using 4 image and clinical data

A model incorporating only three spectrograms and clinical data was used exclusively for blood flow information. It achieved 100% specificity and correctly identified all patients without EAD. However, its sensitivity was 77.1%, which was lower than the 92.4% sensitivity of the ultrasonic-spectrogram fusion network. This suggests that some patients with EAD identified by the ultrasonic spectrogram fusion network could not be identified by the model using only the blood flow information. Therefore, it can be inferred that the EAD identified by the model with three spectrograms and clinical data was caused by an abnormal blood supply, while the remaining EAD identified by the ultrasound spectrogram fusion network was caused by other factors.

### Two-stage radiologists study

#### First phase

In the first phase of the study, six radiologists with varying levels of seniority were recruited to conduct a radiologist-machine comparison without the assistance of artificial intelligence. There were no significant differences in the accuracy, sensitivity, specificity, positive predictive value, or negative predictive value between doctors with high and low seniority in determining whether a patient had EAD based on imaging and clinical data (*P* > 0.050). This suggests that the accuracy of determining EAD is not related to work experience. Notably, the accuracy, sensitivity, and specificity of the less experienced radiologists sometimes exceeded those of the more experienced radiologists. The ultrasonic spectrogram fusion network outperformed the radiologists on all indices by approximately 30%, demonstrating its superior diagnostic performance.

#### Second phase

**Fig. 3:**
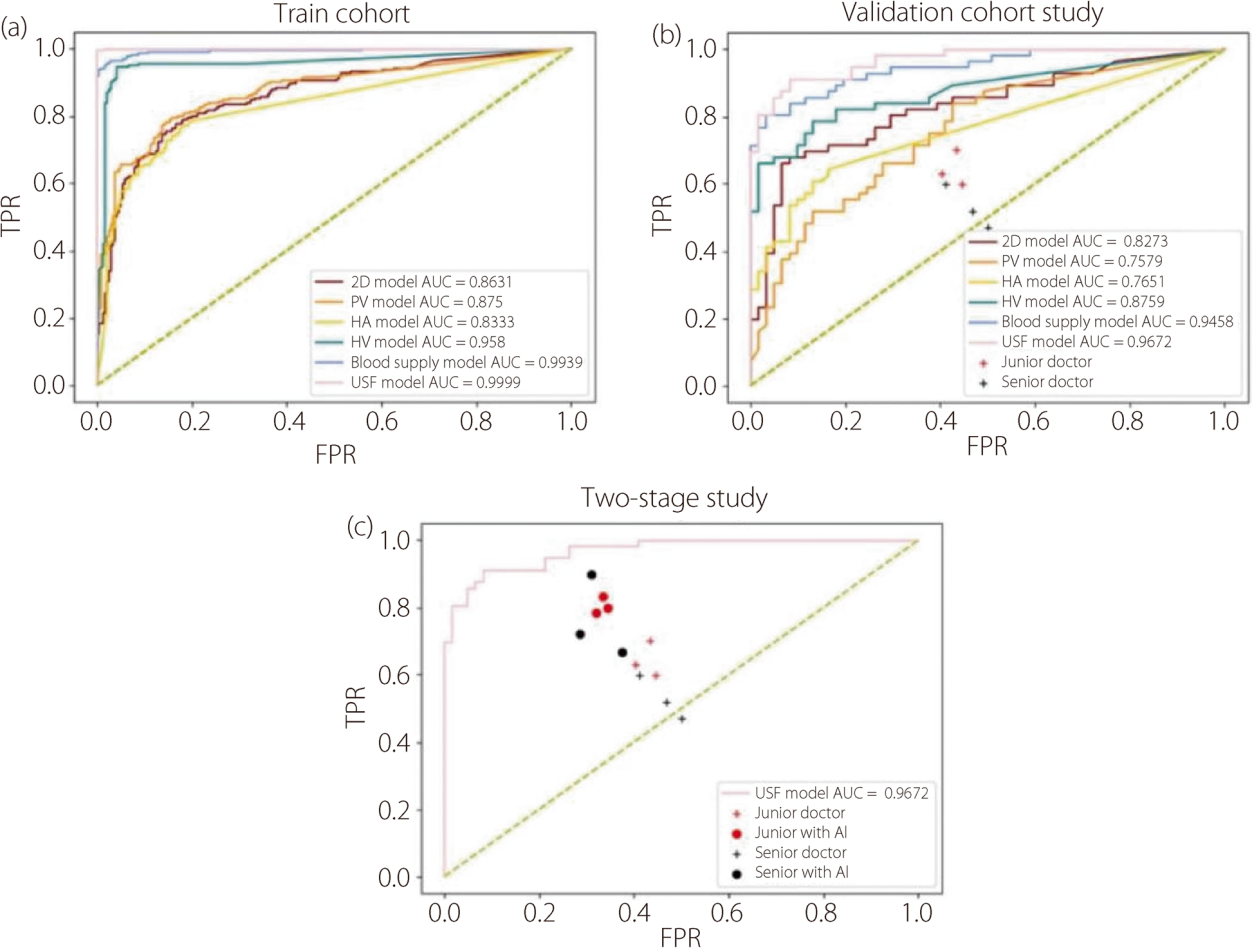
Model and radiologist performance curves (2D model: using grayscale ultrasound image, PV model: using portal vein blood flow spectrum diagram, HA model: using hepatic artery blood flow spectrum diagram, HV model: using hepatic vein blood flow spectrum diagram, blood supply model: using three spectrum and clinical data, USF model: using 4 image and clinical data). **a** Training cohort ROC curve; **b** Validation cohort ROC curve; **c** Two-stage radiologists study ROC curve

In the second phase, with AI-assistance, both senior and junior radiologists showed significant improvements (*P* < 0.05) in accuracy, sensitivity, and specificity. The accuracy increased by 12.2%–16.5%, sensitivity by 16.3%–23.4%, and specificity by 9.5%–13.5%. Although not statistically significant, the positive predictive values increased by 18.3% to 19.2%, and the negative predictive values increased by 14.7% to 21.4%. The benefits of AI-assistance were more pronounced for senior radiologists, while also reducing the disparity in diagnostic performance between radiologists of different seniority levels, thereby narrowing the variances in metrics. Overall, the ultrasound-spectrogram fusion network enhanced the diagnostic precision and consistency of radiologists (Table 3, Fig. 3).

**Table 3:** Before and after study of heatmaps by radiologists

Differrent level of radiologist group		Without→with AI (%)	*P*_1_	*P*_2_	*P*_3_
Junior (*n* = 3)	Accuracy (%)	58.76 ± 2.36→70.94 ± 1.48 ↑	0.206	0.800	0.020
	Sensitivity (%)	64.38 ± 5.30→80.63 ± 2.44 ↑	0.075	0.580	0.004
	Specificity (%)	57.24 ± 2.22→66.73 ± 1.24 ↑	0.340	0.766	0.020
	Positive prediction (%)	32.43 ± 12.68→51.65 ± 6.78 ↑	0.670	0.480	0.230
	Negative prediction (%)	67.08 ± 21.19→88.49 ± 3.03 ↑	0.280	0.390	0.350
Senior (*n* = 3)	Accuracy (%)	53.61 ± 5.43→70.08 ± 5.34 ↑	NA	NA	0.002
	Sensitivity (%)	52.90 ± 6.38→76.30 ± 12.19 ↑	NA	NA	0.008
	Specificity (%)	54.09 ± 4.54→67.63 ± 4.61 ↑	NA	NA	0.003
	Positive prediction (%)	38.76 ± 20.45→57.02 ± 9.96 ↑	NA	NA	0.080
	Negative prediction (%)	67.08 ± 21.19→81.75 ± 11.69↑	NA	NA	0.330

*P*_1_ values indicate a comparison between different radiologist groups without AI assistance

*P*_2_ values indicate a comparison between different levels of radiologist groups with AI-assistance

*P*_3_ values indicate a comparison between different experienced radiologists with AI assistance and senior experienced radiologists without AI assistance

The upward arrow (↑) represents indicators that improved owing to AI assistance

*NA* Not applicable

## Discussion

In this study, we propose an ultrasound-spectrogram fusion network that integrates grayscale ultrasound images, portal vein blood flow spectrograms, hepatic artery blood flow spectrograms, hepatic vein blood flow spectrograms, and clinical data to determine whether a patient is experiencing EAD. This model has been validated to effectively assist radiologists in diagnosing EAD much faster than the laboratory methods widely used in clinical practice. By visualizing their decisions using heat maps, radiologists can learn from these visual aids to improve the diagnosis of EAD, enabling early and rapid screening using ultrasound images and clinical data. To our knowledge, this is the first deep learning-based ultrasound model to analyze EAD from ultrasound images and clinical data involving 580 patients, which ensures the credibility of the study and provides a solid basis for future larger-scale investigations. From a blood flow perspective, our approach offers a novel perspective that may guide clinical treatment strategies and enable more precise and timely interventions for patients with EAD.

Notably, by using the ultrasound spectrogram fusion network, both junior and senior radiologists achieved similarly high accuracy rates, reducing the performance gap between junior and senior radiologists. This demonstrates the network’s potential to help radiologists avoid the subjective biases associated with professional experience, potentially reducing unnecessary investigations and inappropriate or delayed treatments. This is particularly relevant for patients in less developed countries and regions. Regarding model visualization, the heat maps generated by the model can inform doctors better identify the region of interest and avoid missing key areas.

Additionally, existing laboratory tools cannot analyze the causes of EAD, limiting treatment precision and effectiveness. Our model analyzes the portal vein blood flow spectrum, hepatic artery blood flow spectrum, hepatic vein blood flow spectrum, and clinical data to determine whether EAD results from an abnormal blood supply, facilitating targeted therapy.

This study had some limitations. First, the dataset used for model development was sourced from a single medical center. A larger and more diverse dataset is required to develop a more robust model and validate its performance. Second, this study did not address the long-term prognosis of patients with and without EAD. Determining the patient prognosis based on early imaging and data analysis is essential to improving patient survival rates and reducing complications. Finally, the retrospective nature of this study introduced an unavoidable bias. Future studies should incorporate AI systems into routine clinical workflows for prospective validation.

The ultrasound-spectrogram fusion network based on ultrasound images and medical data demonstrated superior accuracy, sensitivity, and specificity compared with radiologists for the early and rapid screening of EAD. It also helps to reduce judgment discrepancies among radiologists, which could be potentially significant for patients in underdeveloped countries and regions. Using only blood flow-related spectrograms and medical data to construct similar models, EAD caused by blood flow abnormalities can be identified, effectively guiding subsequent treatment.

## Conclusions

The ultrasound-spectrogram fusion network offers superior accuracy, sensitivity, and specificity in the early and rapid screening of EAD compared to radiologists, demonstrating its value in clinical practice. The model also reduced diagnostic discrepancies among radiologists, which is crucial for enhancing the quality of care, especially in underdeveloped regions. By focusing on blood flow-related spectra and clinical data, the model accurately identifies EAD cases stemming from blood flow abnormalities and guides appropriate and timely treatments. This study underscores the importance of integrating AI into diagnostic workflows and lays the groundwork for further research and broader clinical adoption to improve patient outcomes.

## Data Availability

The data that support the findings of this study are available from Tianjin First Central Hospital, but restrictions apply to the availability of these data, which were used under the license for the current study and are not publicly available. The data are available from the corresponding author upon request.

## Abbreviations

EAD: Early Allograft dysfunction
AI: Artificial intelligence
LT: Liver transplantation
ROC: Receiver operating characteristic
BMI: Body mass index
MELD: Model for end-stage liver disease
DBCD: Donation after brain and cardiac death
LDLT: Living donor liver transplantation
AUC: Areas under the curve
